# Validation of a mitochondrial RNA therapeutic strategy using fibroblasts from a Leigh syndrome patient with a mutation in the mitochondrial ND3 gene

**DOI:** 10.1038/s41598-020-64322-8

**Published:** 2020-05-05

**Authors:** Yuma Yamada, Kana Somiya, Akihiko Miyauchi, Hitoshi Osaka, Hideyoshi Harashima

**Affiliations:** 1 Faculty of Pharmaceutical Sciences, Hokkaido University, Kita-12 Nishi-6, Kita-ku, Sapporo 060-0812 Japan; 2 Department of Pediatrics, Jichi Medical University, 3311-1 Yakushiji, Shimotsuke, Tochigi 329-0498 Japan

**Keywords:** Molecular biology, Molecular medicine, Materials science, Nanoscience and technology

## Abstract

We report on the validation of a mitochondrial gene therapeutic strategy using fibroblasts from a Leigh syndrome patient by the mitochondrial delivery of therapeutic mRNA. The treatment involves delivering normal ND3 protein-encoding mRNA as a therapeutic RNA to mitochondria of the fibroblasts from a patient with a T10158C mutation in the mtDNA coding the ND3 protein, a component of the mitochondrial respiratory chain complex I. The treatment involved the use of a liposome-based carrier (a MITO-Porter) for delivering therapeutic RNA to mitochondria *via* membrane fusion. The results confirmed that the mitochondrial transfection of therapeutic RNA by the MITO-Porter system resulted in a decrease in the levels of mutant RNA in mitochondria of diseased cells based on reverse transcription quantitative PCR. An evaluation of mitochondrial respiratory activity by respirometry also showed that transfection using the MITO-Porter resulted in an increase in maximal mitochondrial respiratory activity in the diseased cells.

## Introduction

There are currently no available treatments for mitochondrial diseases. The main cause of a mitochondrial disease is a mutation in the mitochondrial DNA (mtDNA). The overall mtDNA encodes for 37 genes, including 13 mRNA molecules that encode for proteins, 22 tRNAs, and 2 rRNAs^1^. Since tRNA and rRNA are involved in mitochondrial transcription and the translation of mtDNA, and all of the 13 proteins that are encoded for are subunits of the respiratory chain complex, mutations in mtDNA can result in serious, life-threatening diseases. Unlike nuclear DNA, there are 2 to 10 copies of mtDNA per mitochondrion^2^, so most cells of individuals with mtDNA mutations are in a heteroplasmy state where both normal mtDNA and mutant mtDNA are simultaneously present^3^. Therefore, a reduction in the mutation rate would be predicted to be an effective treatment strategy.

In order to achieve such a therapy, developing a mitochondrial exogenous expression system that produces therapeutic proteins in mitochondria would be essential. Since the gene codons and transcription/translation systems of these organelles are different from those of nuclei, a specific mitochondrial gene expression DNA vector is needed. Some artificial DNA vectors for mitochondrial gene expression were previously reported, most of which contained genes optimized for mitochondrial endogenous promoters, such as the heavy chain mtDNA promoter (HSP) and the mitochondrial codon system. Some of the previous reports focused on virus vectors resulting in mitochondrial transgene expression first reported by Yu *et al*.^4^. They used an Adeno-associated virus (AAV) as a carrier for achieving mitochondrial gene transfection. The use of AAV is very efficient^5^, but can also result in damage to the immune system, causing infection-related side effects. To circumvent this, we examined the use of a non-viral mitochondrial gene delivery system in an attempt to produce mitochondrial transgene expression by delivering a mitochondrial DNA vector to mitochondria. We used a MITO-Porter system, a liposome-based carrier that is capable of mitochondrial delivery *via* membrane fusion to achieve direct mitochondrial transfection^6,7^.

RNA delivery has recently attracted interest as a gene therapeutic strategy for targeting the nucleus rather than DNA delivery^8–10^. When DNA is delivered to the nucleus, it must be transcribed into mRNA and then translated into a protein, thus requiring both transcription and translation for successful gene expression. When mRNA is delivered, protein is produced only by the translation, and would be expected to be an effective gene therapy with a high gene expression level. In addition, unlike DNA, it would be predicted to not have a sustained effect and would not be expected to induce serious side effects. Concerning targeting the nucleus, research related to DNA delivery was previously a mainstream effort^11–15^, but due to technological innovations, the use of *in vitro* enzyme reactions to produce RNA are now become simpler and less expensive, and many researchers have now focused their research interest on the delivery of RNA^8–10^. Regarding mitochondria, the delivery of DNA and RNA have not been extensively reported, but a procedure involving RNA delivery would be more likely to be useful for the same reason as it is in nuclear-targeted research.

**Figure 1:**
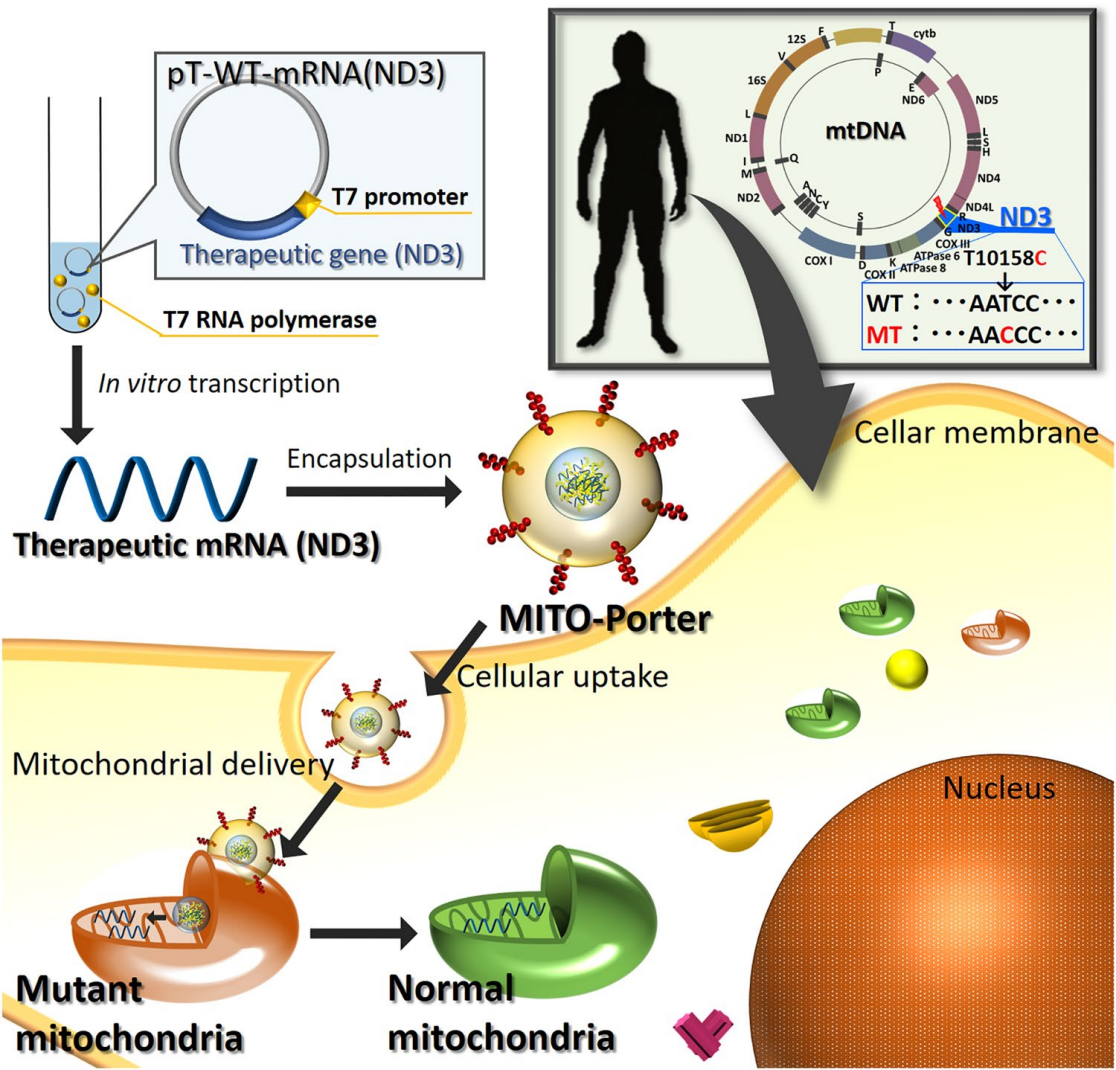
Schematic images of our strategy for mitochondrial RNA therapy targeting mutated mitochondria. As a therapeutic strategy targeting mutated mitochondria, WT-mRNA (ND3) is delivered to mitochondria of fibroblasts derived from a patient with a T10158C heteroplasmic mutation in the mRNA (ND3) of mtDNA in order to decrease the mutant level of the mRNA (ND3) in mitochondria using the MITO-Porter system. Therapeutic WT-mRNA (ND3) was prepared using pT7-WT-mRNA (ND3) with T7 promoter and wild type mRNA (ND3) gene *via in vitro* transcription system and packaged in the MITO-Porter that can deliver cargoes to mitochondria.

In this study, we evaluated a procedure for mitochondrial gene therapy that involves delivering therapeutic RNA to mitochondria using fibroblasts from a patient with a T10158C mutation in the mtDNA that codes for the ND3 protein^16^. The ND 3 protein is a component of the mitochondrial respiratory chain complex I. A large percentage of this point mutation in mtDNA results in a failure to form functional complexes in the mitochondrial respiratory chain, leading to mitochondrial diseases such as Leigh syndrome^17^. In this study, we attempted the mitochondrial delivery of mRNA encoding the wild-type ND3 protein (mRNA (ND3)) to mitochondrial diseased cells, in an attempt to decrease the mutation rate of mRNA (ND3) (Fig. 1). The intracellular trafficking of the MITO-Porter in the diseased cells was first evaluated by determining cellular uptake and intracellular observations. We then transfected the therapeutic mRNA (ND3) into the diseased cells, and the resulting heteroplasmy levels were evaluated by an amplification refractory mutation system (ARMS) - quantitative PCR. We also investigated the therapeutic effect by measuring the mitochondrial respiration of the diseased cells after transfection.

## Results

### Design of the pDNA encoding therapeutic mRNA

**Figure 2:**
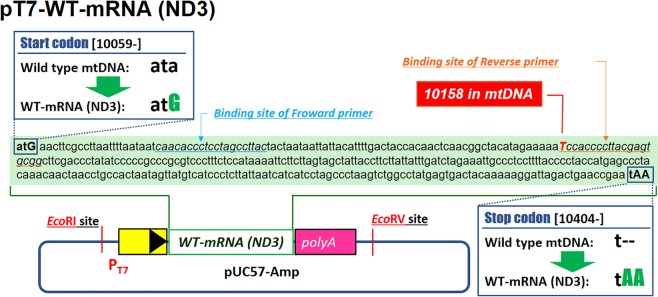
Design of the pT7-WT-mRNA (ND3). pT7-WT-mRNA (ND3) was designed by inserting a DNA fragment containing the T7 promoter and the sequence corresponding to the artificial WT-mRNA (ND3) gene with a poly A sequence (**Sequence S1**) into pUC57-Amp vector between the multi cloning site (*EcoR* I and *EcoR* V sites). We changed from **ata** to **atG** in start codon of WT-mRNA (ND3). We also replaced **tAA** from **t** in the stop codon. The *T* shown *italic* UPPER CASE letter corresponds to base 10158 in the mtDNA coding mRNA (ND3).

In this experiment, therapeutic WT-mRNA (ND3) was prepared using pT7-WT-mRNA (ND3) with a T7 promoter and the wild type mRNA (ND3) gene *via* an *in vitro* transcription system. The therapeutic WT-mRNA (ND3) was designed based on a nuclear transcription/translation procedure, as shown in Fig. 2.

The differences in the native mRNA (ND3) are summarized as follows. Although 10 of the 13 mRNAs encoded by the mtDNA have the start codon ATG, the mRNA (ND3) does not have ATG as start codon. We were concerned that exogenous RNAs that did not undergo transcription/translation from mtDNA might require the ATG sequence as start codon. Thus, we changed ATA to ATG in the start codon of the therapeutic WT-mRNA (ND3). In the case of mRNA transcribed from DNA, the post-transcriptional polyA modification functions as an important factor in the translation process, however it is not known whether the polyA could be modified to the exogenous mRNA delivered to mitochondria. Thus, we hypothesized that RNA sequences with artificially modified polyA would be optimal for translation. In mitochondria, TAA and TAG are recognized as the termination codon, and markers for the end of translation in the mRNA. However, it appears that A is often added later only to T or TA to form TAA. We concluded that the target protein (ND3) would be easier to be translated when T was replaced with TAA. The gene fragments for pT7-WT-mRNA (ND3) were inserted into the restriction enzymes (*EcoR* I and *EcoR* V sites) of the pUC57-Amp vector. The sequence information of these plasmids is summarized in SI (Sequences S1).

### Preparation of the mRNA-MITO-Porter

In this study, we used a MITO-Porter system to deliver therapeutic mRNA to mitochondria. A MITO-Porter was a liposome formulation that can deliver various cargos to mitochondria *via* membrane fusion^18–22^. We previously succeeded in delivering macromolecules such as pDNA in diseased cells using a MITO-Porter, in which pDNA condensed with polycations was encapsulated^6,7^. Similar to packaging pDNA, before encapsulating RNA in the MITO-Porter, nanoparticles of RNA were formed for efficiently encapsulating RNA into the MITO-Porter. A solution of the negatively charged RNA was mixed with a solution of positively charged protamine to form a nanoparticle of RNA *via* electrostatic interactions. We prepared nanoparticles of RNA at a series of nitrogen/phosphate (N/P) ratios, which is the ratio of polycations and RNA, and measured their diameters and ζ-potentials (Fig. S1). In this study, we used small negatively charged particles formed at an N/P ratio of 0.9 (diameter, 145 ± 9 nm; PdI, 0.071 ± 0.01; zeta potential, −35 ± 3 mV, n = 3) for preparing the mRNA-MITO-Porter, because negatively charged particles could be efficiently packaged in MITO-Porter modified with positively charged R8. The RNA nanoparticles were packaged in the MITO-Porter by the ethanol dilution method, the physiochemical properties indicated that the MITO-Porter was a small positively charged carrier that contained encapsulated RNA (diameter, 152 ± 12 nm; PdI, 0.24 ± 0.03; zeta potential, 40 ± 7 mV, encapsulation rate 20 ± 6%, n = 33).

### Intracellular uptake and observations of the mRNA-MITO-Porter

**Figure 3:**
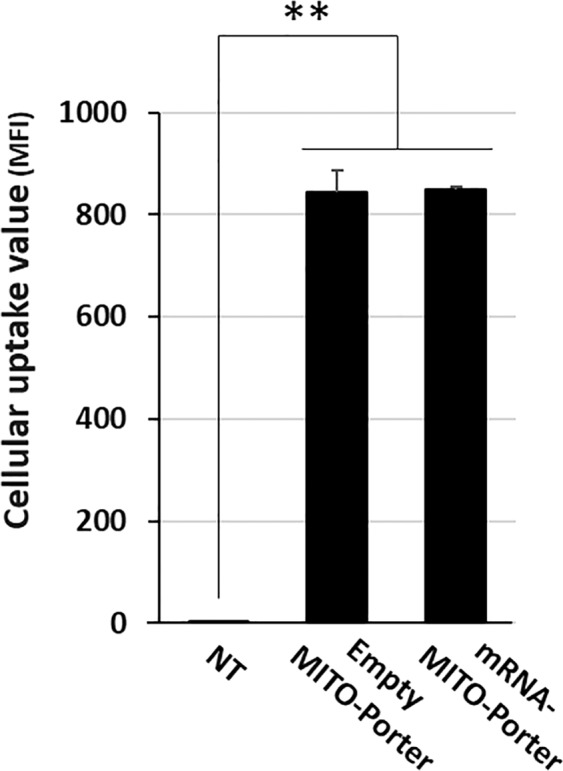
Cellular uptake analysis of the MITO-Porter using flow cytometry. Cellular uptake of the MITO-Porter was evaluated using LS^ND3^ cells. The mean fluorescence intensity (MFI) of the mRNA-MITO-Porter and the empty-MITO-Porter labeled with DiD taken up by cells is shown as values for cellular uptake. In this experiment, 300 ng of RNA was added per well. NT indicates nontreated cells. Data are represented as the mean ± S.D. (n = 3). Significant differences were calculated by one-way ANOVA, followed by the SNK test (**p < 0.01).

**Figure 4:**
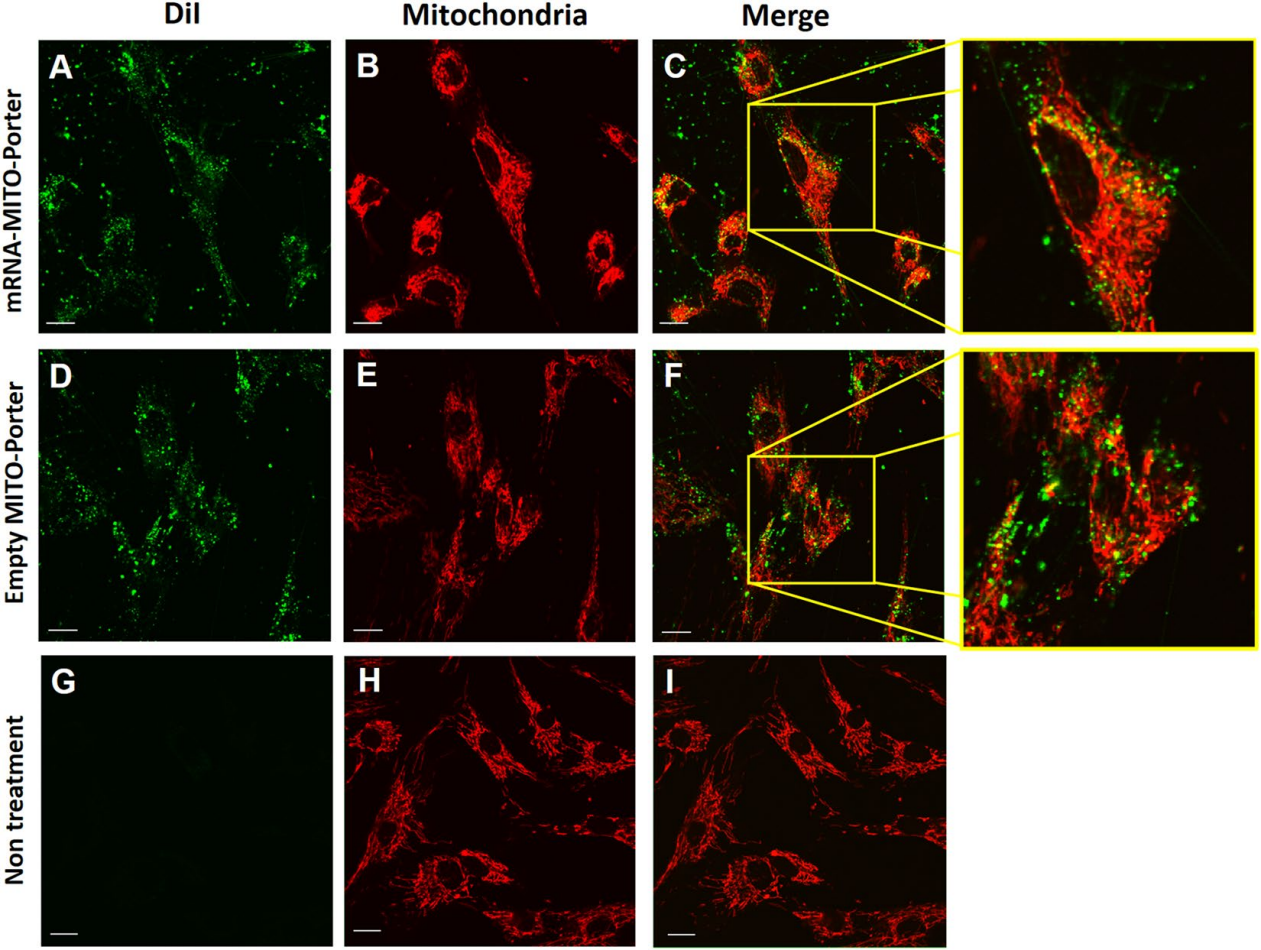
Analysis for intracellular trafficking of MITO-Porter in LS^ND3^ cells. DiI (green fluorescent molecule)-labeled mRNA-MITO-Porter (**A**–**C**) or empty MITO-Porter (**D**–**F**) were incubated with LS^ND3^ cells. In this experiment, 300 ng of RNA was added per dish. Images (**G**–**I**) indicates those of nontreated cells. After staining the mitochondria red with MTDR, the cells were observed by CLSM ((**A**,**D**,**G**), image of mRNA-MITO-Porter; (**B**,**E**,**H**), image of mitochondria; (**C**,**F**,**I**), merge image. Right panels are magnification of marge image. DiI-labeled MITO-Porter appeared as yellow clusters when it was localized in mitochondria. Scale bars; 20 μm.

We investigated the cellular uptake efficiency of mRNA-MITO-Porter labelled with DiD (a fluorescent dye) using LS^ND3^ cells and flow cytometry (Fig. 3). The cellular uptake efficiency of the mRNA-MITO-Porter was comparable to that of the empty-MITO-Porter, indicating that the encapsulated mRNA in the MITO-Porter did not inhibit the cellular uptake process. Intracellular observations also showed that the mRNA-MITO-Porter was taken up by the LS^ND3^ (Fig. 4). In intracellular observations using CLSM, the mRNA-MITO-Porter and the empty MITO-Porter (control) labeled with DiI (pseudo green color) were added to the diseased cells, and the mitochondria were stained red. As a result, numerous yellow dots were observed, indicating that the pseudo green mRNA-MITO-Porters had accumulated in the red stained mitochondria (Fig. 4A–C). In the case of observing the empty MITO-Porter, a similar pattern was observed (Fig. 4D–F). We also calculated the mitochondrial co-localization ratio (Fig. S2) to quantify the co-localization of the MITO-Porter with mitochondria. The evaluation confirmed that the mitochondrial co-localization ratio was comparable between the mRNA-MITO-Porter (16.4 ± 2.7%) and the empty MITO-Porter (16.5 ± 3.0%). These findings confirm that efficiency of mitochondrial accumulation was comparable between the mRNA-MITO-Porter and the empty MITO-Porter.

### Establishment of a method to quantify the mutation rate of mRNA (ND3) in mitochondria

**Figure 5:**
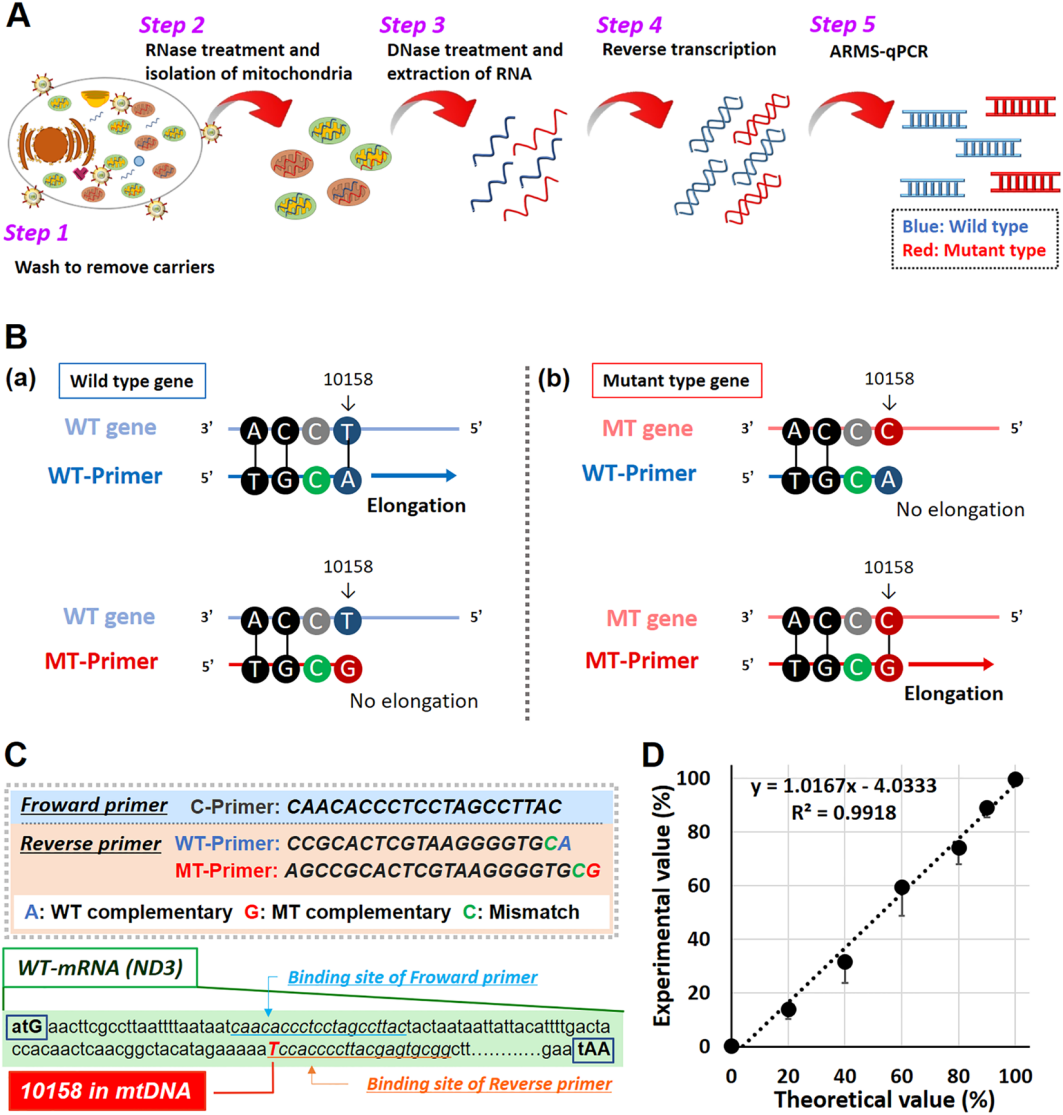
Overview of the method used to quantify the mutation rate of mRNA (ND3) in mitochondria. Schematic protocol for evaluating the mutation rate of mRNA (ND3) in mitochondria after transfection (**A**). The transfected cells were washed in order to remove MITO-Porter that was bound to the surface of cell membranes (Step 1). In the next step, the cells were homogenized, and mitochondria were isolated after RNase treatment to remove RNA absorbed to the surface of mitochondria (Step 2). In the following steps, total RNAs were extracted from the isolated mitochondria (Step 3), cDNAs were prepared using a reverse transcription reaction (Step 4) and ARMS-PCR was then used for the quantitative determination of the mutation rate of mRNA (ND3) (Step 5). Design of reverse primers to distinguish T and C at 10158 in mtDNA coding mRNA (ND3) using ARMS-PCR (**B**). In this experiment, two types of reverse primers including WT primer (−) and MT primer (−) were used. Reverse primers were designed so as to have one mismatches at the 3’ terminal side, and a point mutation (T10158C in mtDNA) was detected. The primers used in this evaluation are shown in Fig. 5C. Standard curve represents the relationship between theoretical value (X-axis) and experimental value (Y-axis) of mixed ratio of wild-type and mutant pDNA (**D**).

As shown in Fig. 5A, mitochondrial RNA was extracted from the LS^ND3^ cells and was changed to cDNA *via* reverse transcription, followed by quantification of the mutation rate of the mRNA (ND3) using the ARMS-PCR method. Briefly, the transfected cells were washed with CellScrub buffer to remove MITO-Porters that were bound to the surface of cell membranes (Step 1). In the next step, the cells were homogenized, and mitochondria were isolated after an RNase treatment to remove RNA that was absorbed to the surface of the mitochondria (Step 2). In the following steps, total RNAs were extracted from the isolated mitochondria (Step 3), cDNAs were prepared using a reverse transcription reaction (Step 4) and ARMS-PCR was then used for the quantitative determination of the mutation rate of mRNA (ND3) (Step 5).

The ARMS-PCR method is generally used to detect mutations in the nuclear genome^23^, but has also recently been used as a method for detecting the mutation rate of mtDNA^24^. In this experiment, a common forward primer (C primer) binding to the sequence of the mRNA (ND3) gene in mtDNA (10085–10104 bases) and two types of reverse primers including a WT primer and an MT primer were used. Reverse primers were designed so as to contain one mismatch at the 3’ terminal side, and a point mutation (T10158C in mtDNA) was detected (Fig. 5B). For example, the WT primer can detect a wild-type gene because the C in the 3’ terminal of WT primer can bind to 10158 T in a wild-type gene resulting in elongation, while the MT primer can bind to 10158 C in the mutant gene (Fig. 5B). The primers used in this evaluation are shown in Fig. 5C. The binding site of the primers on mtDNA is shown in Fig. 2. The mutation rate was calculated using the formula (1), as shown in the Experimental Section. In order to design the optimal primer sets, pDNA encoding the target wild-type gene and the mutant gene (pT7-WT-mRNA (ND3), pT7-MT-mRNA (ND3)) were mixed at a ratio of 0–100%, and quantitative ARMS-PCR was performed using various types of primers. As a result, an ideal standard curve which is the theoretical value (X axis) the experimental value (Y axis) was almost same (slope is ~1) with a high accuracy using the primer sets shown in Fig. 5C (Fig. 5D).

To verify the validity of this primer set, the mutation rate of the mRNA (ND3) in LS^ND3^ cells was measured using the primer set shown in Fig. 5C, and it was found that the mutation rate was about 80%. This value was close to the 90% reported as the mtDNA mutation rate in a previous study^16^. Moreover, the mutation rates of NB1RGB cells (normal fibroblasts), which do not contain mutant mRNA (ND3), were calculated to be 0%. These results indicate that the quantitative ARMS-PCR method using a designed primer set, which was optimized in terms of the length and number of mismatch bases, can be used to evaluate the mutation rate of mRNA (ND3).

### Evaluation of the mutation rate of WT-mRNA (ND3) in mitochondria of LS^ND3^ cells after the mitochondrial transfection of therapeutic mRNA

**Figure 6:**
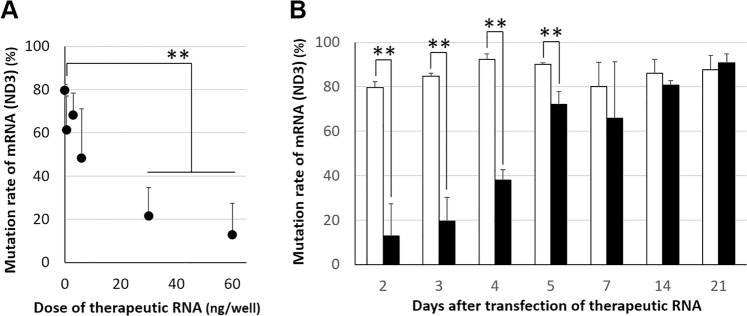
Evaluation of mutation rate of mRNA (ND3) in mitochondria of LS^ND3^ cells after transfection. LS^ND3^ cells were transfected with therapeutic RNA (WT-mRNA (ND3)) using MITO-Porter system. (**A**) Mutation rates were measured at 2 days after the transfection of different doses of the pre-WT-mRNA (ND3) [0.6, 3, 6, 30 and 60 ng/well]. Data represent the mean ± S.D. (n = 3). Significant differences (**p < 0.01) were calculated by Non-repeated ANOVA followed by the Bonferroni test (vs nontreatment). (**B**) Mutation rates were measured at 2–21 days after the transfection of the WT-mRNA (ND3) (60 ng/mL). Open and closed bars represent nontreatment and transfection groups, respectively. Data represent the mean ± S.D. (n = 3–6). Significant differences were calculated by student t-test (**p < 0.01).

We investigated the issue of whether the mitochondrial transfection of WT-mRNA (ND3) into LS^ND3^ cells by the MITO-Porter could reduce the mutation rate of the mRNA (ND3). The mutation rates were measured by the quantitative ARMS-PCR method, at 48 hrs after the transfection of different doses of the pre-WT-mRNA (ND3) (Fig. 6A). The mutation rates were calculated to be about 10% at a dose of 60 ng/well and about 20% at a dose of 30 ng/well, and were significantly reduced compared to about 80% for non-treated cells. These results indicate that the mutation rates decreased depending on the doses being used, when therapeutic mRNA is transfected with the MITO-Porter.

Furthermore, when the mutation rates were evaluated for up to two weeks after the transfection, the state in which the mutation rate was reduced was maintained after a single transfection of mRNA-MITO-Porter (60 ng/mL). As shown in Fig. 6B, the findings confirmed that the mRNA-MITO-Porter transfection group maintained a significantly lower mutation rate than the non-treated cell group for up to 5 days after the transfection.

### Evaluation of mitochondrial function after the mitochondrial transfection of therapeutic mRNA using the MITO-Porter system

Respiration, which consumes oxygen and produces energy, is one of important functions of mitochondria. The ND3 protein that was the focus of this study is a protein constituting the respiratory chain complex I responsible for respiration. Thus, it was expected that overall respiratory ability would increase when the mitochondrial transfection of WT-mRNA (ND3) was achieved in LS^ND3^ cells which contain mutant mRNA coding for ND3. In our previous report^25^, it was confirmed that the respiratory ability of LS^ND3^ cells was significantly decreased compared that of normal cells. In particular, a remarkable decrease in respiratory activity related to complex I was observed. Therefore, we concluded that the LS^ND3^ cells were a suitable model diseased cell to verify a therapeutic effect by the mitochondrial delivery of therapeutic mRNA coding ND3 related to complex I.

**Figure 7:**
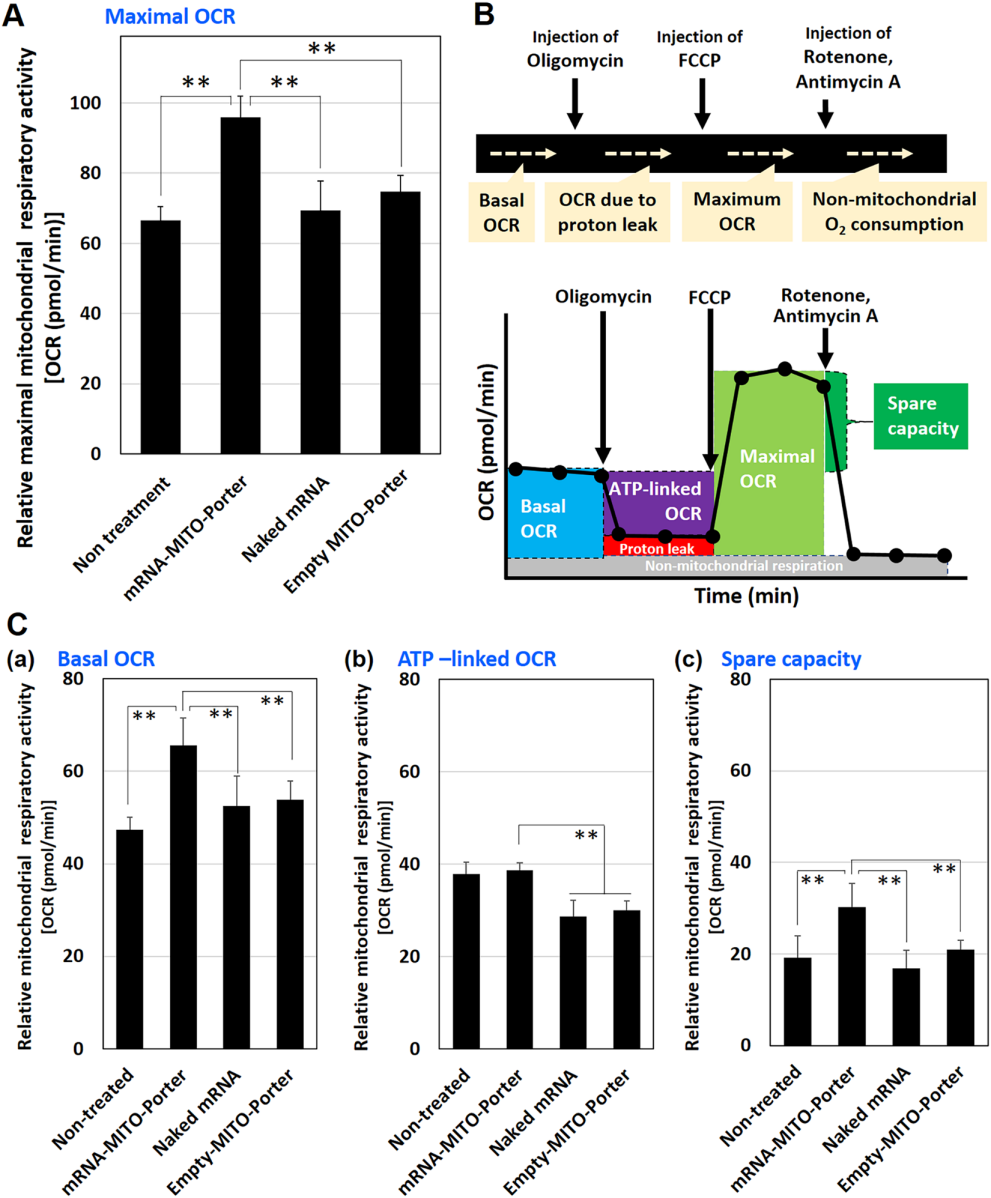
Evaluation of mitochondrial respiratory activities after transfection of therapeutic mRNA. (**A**) LS^ND3^ cells were transfected with therapeutic RNA (WT-mRNA (ND3)) using the MITO-Porter system (60 ng/mL) and maximal mitochondrial respiratory activity [maximal OCR] was measured at 72 hr after the transfection. (**B**) Protocols used for assessing mitochondrial respiratory activities by measuring the OCR. The OCR values were measured after the sequential injection of oligomycin (to inhibit the action of ATP synthase), FCCP (to uncouple the mitochondrial inner membrane and allow for maximum electron flux through the electron transport chain), rotenone (to inhibit complex I) and antimycin A (to inhibit complex III). (**C**) Relative mitochondrial respiratory activities regarding basal OCR (a), ATP-linked OCR (b) and spare capacity (c) are shown. We also compared the value with naked therapeutic mRNA and empty MITO-Porter. Data are the mean ± S.D. (n = 10–15). Significant differences (**p < 0.01) were calculated by Non-repeated ANOVA followed by Bonferroni test (vs mRNA-MITO-Porter).

After the mitochondrial transfection of the therapeutic mRNA using the MITO-Porter system (60 ng/mL), the maximal oxygen consumption rate (OCR) to estimate relative maximal mitochondrial respiratory activities was measured (Fig. 7A). The protocols used for assessing the mitochondrial respiratory activities by measuring the OCR are shown in Fig. 7B. The OCR values were measured after the sequential injection of oligomycin (to inhibit the action of ATP synthase), carbonyl cyanide p-trifluoromethoxy-phenyl-hydrazone (FCCP) (to uncouple the mitochondrial inner membrane and allow for maximum electron flux through the electron transport chain), rotenone (to inhibit complex I) and antimycin A (to inhibit complex III). The maximal OCRs, which are the difference in the values of the OCRs before and after the injection of FCCP were evaluated as maximal mitochondrial respiratory activity. As a result, the maximal mitochondrial respiratory activity caused by the treatment with the mRNA-MITO-Porter was significantly increased compared to those treated with the empty MITO-Porter, naked therapeutic mRNA (Fig. 7A).

We also evaluated other mitochondrial respiratory activities regarding basal OCR, ATP-linked OCR and spare capacity (Fig. 7C). In the evaluation of the spare capacity regarding, the OCR of LS^ND3^ cells significantly increased by transfection with mRNA-MITO-Porter (Fig. 7C(c)) as in the case of the maximal mitochondrial respiratory activity (Fig. 7A). In addition, the OCR regarding basal respiration was significantly higher in the mRNA-MITO-Porter transfection group (Fig. 7C(a)). These results indicate that mitochondrial function is increased by the mitochondrial delivery of therapeutic WT-mRNA (ND3) to LS^ND3^ cells using the MITO-Porter. On the other hand, the ATP-linked OCR after a treatment with oligomycin to inhibit complex V were comparable between the mRNA-MITO-Porter treatment and the non-treatment (Fig. 7C(b)). This result suggests that the mitochondrial delivery of WT-mRNA (ND3) coding the ND3 protein related to complex I would not affect the OCR related to complex V.

## Discussion

The development of an effective therapy for the treatment of mitochondrial diseases is needed, and many clinical trials have been conducted in attempts to achieve this^26^. On the other hand, very few drugs for the treatment of mitochondrial diseases have been approved to date. In Europe, only Raxone (Idebenone) is approved for Leber’s hereditary optic neuropathy (LHON)^27,28^, and in Japan, Taurine is approved for mitochondrial myopathy, encephalopathy, lactic acidosis, and stroke-like episodes (MELAS)^29^. Idebenone, taurine and most therapeutic candidates are small molecule drugs that attempt to temporarily improve mitochondrial dysfunction. The main cause of mitochondrial diseases such as MELAS, however, is gene mutation^30^. Thus, mitochondrial gene therapy is required for radical treatment.

In this study, we investigated a mitochondrial gene therapeutic strategy that targets a point mutation (T10158C) in the ND3 protein coding region in the mtDNA. Some studies, mainly using allotopic expression such as a gene therapeutic strategy have been reported, but the successful examples of this strategy are only ATP6/8 and ND1, 4 genes among the 13 proteins encoded by mtDNA^31–34^. Thus, this strategy has not been applied to all mitochondrial proteins that include the ND3 protein. Although ~99% of genes coding proteins that are localized in mitochondria were transferred to nuclear DNA during evolution, only 1% of these proteins are conserved in the mtDNA^35^. This suggests that these proteins expressed in the nucleus are transported to mitochondria with difficulty. Therefore, direct mitochondrial transfection would be a useful tool for achieving mitochondrial gene therapy. To address this, we examined the issue of whether mitochondria function would be restored by the direct mitochondrial transfection of wild type mRNA (ND3) to mitochondria of LS^ND3^ cells, where mutant ND3 proteins that decrease mitochondria function are present. It was assumed that wild type ND3 protein could be produced from the therapeutic mRNA inside mitochondria.

In this study, a MITO-Porter modified with R8 (a cationic peptide) was used for mitochondrial transfection, and it was presumed that the positively charged MITO-Porter would be able to access negatively charged mitochondria *via* the electrostatic interactions^18,19^. Although, the mitochondrial respiratory activities of LS^ND3^ cells were decreased^25^, it was confirmed that the mitochondrial membrane potential remained to some extent (data not shown). Therefore, we concluded that the mitochondrial accumulation of R8-MITO-Porter in LS^ND3^ cells would be contributed by the electrostatic interactions between the MITO-Porter and mitochondria. As a result, the value for the co-localization of the MITO-Porter with mitochondria was less than 20% (Fig. S2). We previously succeeded in the mitochondrial delivery of functional cargoes using an R8-modified MITO-Porter to regulate mitochondrial function^21,36^. In these studies, it was also confirmed that some of the MITO-Porters accumulated in mitochondria and others were located in other cellular compartments, although, a carrier with no mitochondrial targeting ability (other than the MITO-Porter) was ineffective. These findings suggest that certain amounts of molecules can be delivered to mitochondria.

As a result of evaluating the mutation rate after treatment with the mRNA-MITO-Porter, it was confirmed that wild type mRNA was, in fact, delivered to mitochondria, and the mutation rate was reduced and this was maintained for about one week or less (Fig. 6). It is expected that this will be useful as a medicine that will lower the mutation rate, that this rate will be maintained for several days and that the effect is reversible. This is because it does not require frequent administration that lowers QOL and does not induce serious side effects due to transient effects. In addition, a significant reduction in the mutation rate and recovery of respiratory ability were observed at a dose of 60 ng RNA/well (Fig. 6). This is significantly less than the amount of idebenone and taurine, which was needed to be effective in clinical trials, respectively (1/50 or less)^37,38^. This point is also relevant when practical use is being considered.

The findings shown in Fig. 7 confirm that the mitochondrial transfection of therapeutic mRNA coding wild type ND3 protein using the MITO-Porter system led to the recovery of mitochondrial respiratory activity. Mitochondrial respiratory chain complexes that are responsible for mitochondrial respiration range from I to IV and V (ATP synthetase), and respiratory chain complexes I, III, and IV form super complexes, thus allowing mitochondrial electron transfer for respiration to occur^39,40^. The ND3 protein is one of the proteins that make up the respiratory chain complex I. As a result, mutations in the ND3 protein would cause a decrease in mitochondrial respiration due to the abnormal formation of the respiratory chain complex I and super complex formation^41,42^. The findings reported herein serve to confirm that the mitochondrial transfection of WT-mRNA (ND3) resulted in the production of the normal ND3 protein from the delivered mRNA and improved mitochondrial respiration in diseased cells. The result led us to the assumption that the ND3 protein produced from the delivered mRNA might contribute to the formation of normal respiratory chain complex I, thus improving the mitochondrial respiration of diseased cells.

Several small sized drugs are approaching practical use as effective mitochondrial treatments, but other therapeutic strategies including mitochondrial gene therapy are being actively investigated. The gene therapy strategy includes the allotopic expression and direct mitochondrial transfection with a virus vector. This study represents the first successful verification of a novel mitochondrial gene therapeutic strategy for delivering wild type mRNA coding for the ND3 protein as a therapeutic drug directly to mitochondria using non-viral vector. Our findings suggest that our mitochondrial transfection system could be also useful for mRNA molecules in addition to mRNA (ND3), and therefore has the potential for expanding the treatment of various mitochondrial diseases that are caused by a mutation in the mitochondrial genome. We hope that this study will be the first step towards making mitochondrial diseases a “cured disease”.

## Methods

The experiment protocol using fibroblasts from a Leigh syndrome patient was approved by the ethics boards of Jichi Medical University (Genetics 18-07) and the Faculty of Pharmaceutical Sciences, Hokkaido University (No. 2017–005). All experiments were performed in accordance with relevant guidelines and regulations. Written informed consent was obtained from either or both parents of the patient.

### Materials

1,2-dioleoyl-sn-glycero-3-phosphatidyl ethanolamine (DOPE) and sphingomyelin (SM) were obtained from Avanti Polar lipids (Alabaster, AL, USA). Protamine was purchased from CALBIO CHEM (Darmstadt, Germany). Stearylated octaarginine (STR-R8)^43^ was obtained from KURABO Industries Ltd (Osaka, Japan). Lipofectamine iMRX (LFN iMax), 1,1′-dioctadecyl-3,3,3′,3′-tetramethylindodicarbocyanine perchlorate (DiD), 1,1′-dioctadecyl-3,3,3′,3′-tetramethylindocarbocyanine perchlorate (DiI) and MitoTracker Deep Red (MTDR) were purchased from Thermo Fisher Scientific Life Sciences (Waltham, MA, USA). Dulbecco’s modified Eagle’s medium (DMEM) with a low glucose content was purchased from Gibco (Massachusetts, USA). Fetal bovine serum (FBS) was purchased from Sigma-Aldrich Co. LLC. (Roche; Darmstadt, Germany). Purified oligonucleotides were purchased from Sigma Genosys Japan (Ishikari, Japan). All other chemicals used were commercially available reagent-grade products.

### Cell culture

LS^ND3^ cells^16^ were obtained from a patient with Leigh syndrome with a T10158C point mutation in the mtDNA at the Jichi Medical University. The mitochondrial DNA in the LS^ND3^ cells of this patient contained a heteroplasmic mutation in the ND3 protein. The ND3 protein is a component of complex I. A high mutation rate causes Leigh syndrome, a neurodegenerative disease the develops in infancy and childhood^44^. The LS^ND3^ cells were maintained in DMEM with a low glucose level that contained 10% FBS, supplemented with penicillin and streptomycin. The cells were grown in 10 cm dishes at 37 °C under an atmosphere of 5% CO_2_. Cells were passed on every 4–7 days.

### Design and preparation of pDNA encoding therapeutic mRNA expressing wild-type ND3

We designed artificial pDNA vector as described in the experimental section of the paper we previously reported^45^. The gene fragments for pT7-WT-mRNA (ND3) was synthesized by GENEWIZ (South Plainfield, NJ, USA), and the synthesized genes were inserted into the restriction enzymes (*EcoR* I and *EcoR* V sites) of the pUC57-Amp vector (GENEWIZ). pT7-MT-mRNA (ND3) was constructed by inserting a point mutation (T - > C), according to the manufacturer’s recommended protocol using a PrimeSTAR Mutagenesis Basal Kit (TAKARA BIO INC, Shiga, Japan) with 5′-agaaaaa**C**ccaccccttacgagagtgcg -3′ (forward) and 5′-ggggtgggtttttctatgtagccgtt-3′ (reverse). The base with bold capital indicates mutation point in mtDNA (T10158C). The sequence information of these plasmids is summarized in SI (Sequences S1–S2). The pDNA used in this experiment were amplified in E. coli strain DH5α, and purified using an Endofree Plasmid Midi Kit (Qiagen GmbH, Hilden, Germany).

### Purification of the RNA *via in vitro* transcription system

RNAs were prepared using template DNAs *via* an *in vitro* transcription system including T7 RNA Polymerase. In a typical run, mRNA (ND3) was transcribed from linear DNA of pT7-WT-mRNA (ND3) by RiboMAX Large Scale RNA Production System (Promega Corporation, Madison, WI, USA) according to the manufacturer’s recommended protocol for T7 promoters. The linear DNA was prepared by digestion of the pT7-WT-mRNA (ND3) with *EcoR* V and the resulting material was purified by phenol/chloroform extraction and ethanol precipitation. The synthesized RNAs were purified by Nucleospin RNA Cleanup (MACHEREY-NAGEL GmbH & Co. KG, Duren, Germany) according to the manufacturer’s recommended protocol. The quality of the purified RNA was measured using a NanoDrop (ND-2000; Thermo Fisher Scientific, Inc.).

### The packaging of therapeutic mRNA in MITO-Porter

The mRNA-MITO-Porter was constructed by the ethanol dilution method. mRNA nanoparticles at an N/P ratio of 0.9 were prepared by titrating 75 μL of mRNA (0.3 mg/mL) into 75 μL of protamine (0.18 mg/mL), a cationic peptide, while mixing in 10 mM HEPES buffer. The resulting mRNA nanoparticles were encapsulated in lipid membranes by adding 65 µL of a nanoparticle suspension to 108 µL of a lipid solution containing DOPE/SM/STR-R8 (molar ratio: 9/2/1) in 100% EtOH, and the solution was added to 630 µL of HEPES buffer under vortexing. When the empty-MITO-Porter was prepared, a HEPES buffer instead of the nanoparticle suspension was added to the lipid solution. After diluting the resulting suspension with 4 mL of HEPES buffer, the ethanol was removed by ultrafiltration using Amicon Ultra-4-100K filter (Merck KGaA; Merck Millipore, Darmstadt, Germany), and the retained solution was again diluted with 4 mL of HEPES buffer to replace the ethanol buffer to the HEPES buffer. The diluted ethanol solution was again removed, resulting in the complete formation of the mRNA-MITO-Porter.

We evaluated physicochemical properties of the carriers used in this study as described in the experimental section of the paper we previously reported^46^. Parts of the section are written below. Particle diameters and polydispersity index (PDI) (an indicator of particle-size distribution) were measured by dynamic light scattering (DLS) (Zetasizer Nano ZS; Malvern Instruments, Worcestershire, UK). Samples were prepared in 10 mM HEPES buffer at 25 °C and the values for particle diameters are shown in the form of volume distribution. The ξ-potentials of the samples were also determined in 10 mM HEPES buffer at 25 °C using a Zetasizer Nano ZS.

### Evaluation of the cellular uptake of the mRNA–MITO-Porter by flow cytometry

The empty-MITO-Porter and the mRNA-MITO-Porter, which were labelled with 1 mol% DiD, were prepared by the ethanol dilution method. At 24 hrs before the experiment, cells (5 × 10^4^ cells/well) were seeded in 6-well plates (Becton, Dickinson and company (Corning), Franklin Lakes, NJ, USA), and the resulting plates then incubated at 37 °C under 5% CO_2_. After washing the cells, they were incubated with DMEM (FBS-) containing MITO-Porters for (300 ng RNA) 1 hr. To remove the MITO-Porters from the medium, the cells were washed once with Phosphate buffered saline (PBS) and twice with a heparin solution (20 U/mL in PBS). Cells were collected by trypsinization and suspended in 1 mL of FACS Buffer (PBS containing 0.5% bovine serum albumin (BSA) and 0.1% NaN_3_). The resulting suspension was centrifuged and resuspended in 0.5 mL of FACS Buffer. After filtering through a nylon mesh, cells were analyzed by FACS Galios (Beckman coulter, Inc., California, USA). The procedure involved excitation with a 638 nm light, and the fluorescence detection channel was set to a 660 nm FL6 filter for detection of DiD.

### Intracellular observation of the MITO-Porter by CLSM

The empty-MITO-Porter and mRNA-MITO-Porter, labelled with 0.1 mol% DiI, were prepared by the ethanol dilution method. At 24 hrs before being observed, the cells (5 × 10^4^ cells) were seeded in 35 mm glass-bottom dishes (AGC TECHNO GLASS CO., LTD. (IWAKI), Shizuoka, Japan) at 37 °C under 5% CO_2_. After washing the cells twice with 1 mL of DMEM (FBS-), they were incubated with DMEM (FBS-) that contained the MITO-Porters (300 ng RNA). After a 1 hr period of incubation, the medium was removed and 1 mL of DMEM (FBS+) was added. After the cells were incubated for 1 hr and 40 min, DMEM was then removed, and the cells were stained with MTDR in DMEM (1 mL, final concentration of 100 nM) for a further 20 min. After washing the cells with 1 mL DMEM (phenol red -), 2 mL of DMEM (phenol red -) was added. Cells were analyzed by CLSM using FV10i-LIV (Olympus Corporation, Tokyo, Japan), based on the experimental section of the paper we previously reported^47^. The cells were excited with a 559 nm light from an LD laser. Images were obtained using an FV10i-LIV equipped with a water-immersion objective lens (UPlanSApo 60×/NA. 1.2) and a dichroic mirror (DM405/473/559/635). The two fluorescence detection channels (Ch) were set to the following filters: Ch1: 570/50 (red) for DiI, Ch2: 660/50 (red) for MTDR.

### Quantification of the mutation rate of mRNA by ARMS-PCR

At 24 hrs before the experiment, cells were seeded in a 6-well plate (1 × 10^5^ cells/well) or a 10 cm dish (6 × 10^5^ cells/dish) at 37 °C under an atmosphere of 5% CO_2_. After washing the cells, they were incubated with DMEM (FBS-) containing mRNA-MITO-Porter. The cells were transfected with mRNA using LFN iMax according to the manufacturer’s recommended protocol. After 3 hrs, the medium was replaced with DMEM (+FBS), and the resulting suspension further incubated at 37 °C under an atmosphere of 5% CO_2_. After the incubation, cells were washed with PBS and, collected by trypsinization. the collected cells were also suspended in Cell scrub buffer (Genlantis Inc., San Diego, Calif., USA) and incubated for 15 min at 37 °C to remove the MITO-Porters that were attached to the surfaces of the cells, followed by centrifugation at 700 *g* for 3 min at 4 °C. We then extracted mitochondrial RNA from the LS^ND3^ cells and reverse transcribed to obtain cDNA as described in the experimental section of the paper we previously reported^45^. Parts of the section are written below.

The cells were centrifuged again and the buffer replaced with a mitochondrial isolation buffer (MIB) [250 mM sucrose, 2 mM Tris–HCl, 1 mM EDTA, pH 7.4]. The resulting suspension was homogenized with a 27-gauge needle and the resulting homogenate centrifuged at 700 *g* for 10 min at 4 °C to remove the fraction containing nuclei and cell-derived debris. The supernatant, that contained mitochondria, was treated with RNase to remove RNA exterior to the mitochondria and centrifuged at 700 *g* for 10 min at 4 °C. The resulting supernatant was added to a 60% percoll solution (GE Healthcare UK, Ltd., Buckinghamshire, UK) and centrifuged at 20,400 g for 10 min at 4 °C. The mitochondria that were located at the interface between the percoll solution and EDTA-free MIB [250 mM sucrose, 2 mM Tris–HCl, pH 7.4] were collected, and, after adding 650 µL of EDTA-free MIB, the suspension was centrifuged at 20,400 g for 15 min at 4 °C. After removing the supernatant, 500 µL of EDTA-free MIB was added to the pelleted fraction and the suspension was again centrifuged at 20,400 g for 15 min at 4 °C. The supernatant was removed and isolated mitochondria were obtained in pelleted form. Total RNAs in mitochondria were extracted using an RNeasy Mini Kit (Qiagen, Hilden, Germany) according to the manufacture’s protocol, combined with DNase I digestion for the degradation of DNA in total RNA samples using RNase-Free DNase Set (QIAGEN). Obtained RNAs were reverse transcribed by High Capacity RNA-to-cDNA kit (Thermo fishier Scientific (Applied Biosystems)), according to the manufacturer’s protocol.

A quantitative ARMS-PCR analysis was performed on the cDNA using the THUNDERBIRD SYBR qPCR Mix (TOYOBO CO., LTD., Osaka, Japan) and LightCycler 480 (Sigma-Aldrich Co. LLC. (Roche)). All reactions involved the use of a volume of 5 μL. In this experiment, common forward primer (C primer) binding to sequence of ND3 gene in mtDNA corresponding to 10085-10104 bases was used, and the reverse WT primer for recognition of the 10158 T in the sequence of the ND3 gene in the mtDNA (wild type) and reverse MT primer for recognition to 10158 C in the sequence of the ND3 gene in the mtDNA (mutant) were designed. Reverse primers were designed so as to contain one mismatch at the 3’ terminal side, and a point mutation (T10158C in mtDNA) was detected. Threshold cycles (C_T_) of the wild type gene and the mutant gene were measured and the mutation rate was calculated using formula (1) as described below:

1 $${\rm{Mutation}}\,{\rm{rate}}\,{\rm{of}}\,{\rm{mRNA}}({\rm{ND3}})\,( \% )=\frac{1}{1+{\left(\frac{1}{2}\right)}^{\Delta {C}_{T}}}\times 100$$

### Evaluation of mitochondrial respiratory capacity

At 24 hrs before the experiment, cells (2 × 10^5^ cells/well) were seeded on a 60 mm dish. After removing the medium, 2 mL of DMEM (−) containing mRNA-MITO-Porter (60 ng RNA/mL) was added to the cells in each dish, followed by incubation for 3 hr at 37 °C under an atmosphere of 5% CO_2_. The medium was replaced with DMEM (+FBS), and again incubated at 37 °C, 5% CO_2_ for 45 hr. After the incubation, each cell (2.0 × 10^4^ cells/80 μL/well) were seeded in a 96-well plate, and incubated for 24 hr at 37 °C under 5% CO_2_. We evaluated mitochondrial respiratory capacity as described in the experimental section of the paper we previously reported^25^. Parts of the section are written below. Respiratory activity was determined by measuring the values for the OCR using a Seahorse XFe 96 extracellular flux analyzer (Agilent Technologies, Santa Clara, CA, USA). Prior to the analysis, the culture medium was replaced with assay medium, and after allowing the solution to equilibrate for 1 hr at 37 °C in the absence of CO_2_, the OCR values were measured after the sequential injection of oligomycin (2 μM), FCCP (2 μM), rotenone (0.5 μM) and antimycin A (0.5 μM) to determine the OCR to evaluate maximal respiratory activity (Fig. 7A), and mitochondrial respiratory activities regarding basal OCR, ATP-linked OCR and spare capacity (Fig. 7B), as previously reported^25^. We examined 10 wells per experiment in a 96-well plate. We used the normalization function of an Agilent Seahorse XF assay to account for variations in the DNA content as a relevant cell parameter,following the manufacturer’s recommended protocol. The DNA content was examined by a CyQUANT Cell Proliferation Assay Kit (Invitrogen, Thermo Fisher Scientific Life Sciences).

### Statistical analysis

For comparisons of the two groups, the statistical significance was calculated by the Student’s t-test. For multiple comparisons, one way ANOVA was performed, followed by the Bonferroni/Dunn test. Levels of P < 0.05 were considered to be significant.

## Supplementary information

Supplementary information.
