# Supplementary material for: Validation of a mitochondrial RNA therapeutic strategy using fibroblasts from a Leigh syndrome patient with a mutation in the mitochondrial ND3 gene
Source: Sci Rep. 2020 May 5;10:7511. doi: 10.1038/s41598-020-64322-8 (PMC7200808; doi:10.1038/s41598-020-64322-8)
Supplement: Supplementary file 1 — Supplementary information. [file 41598_2020_64322_MOESM1_ESM.pdf]

Supplemental Information (SI)

Validation of a mitochondrial RNA therapeutic strategy using fibroblasts from a Leigh syndrome patient with a mutation in the mitochondrial ND3 gene

Yuma Yamada[1,*], Kana Somiya[1], Akihiko Miyauchi[2], Hitoshi Osaka[2] and Hideyoshi Harashima[1,]**

[1]Faculty of Pharmaceutical Sciences, Hokkaido University, Kita-12 Nishi-6, Kita-ku, Sapporo 060-0812, Japan
[2]Department of Pediatrics, Jichi Medical University, 3311-1 Yakushiji, Shimotsuke, Tochigi, 329-0498, Japan
[*]u-ma@pharm.hokudai.ac.jp, [**]harasima@pharm.hokudai.ac.jp

Materials and methods

RNA extraction for quantification of the mutation rate of mRNA (ND3)

A schematic protocol for this study is shown in Figure 5A. After transfection, the cells were washed with PBS (-), and then PBS (-) containing heparin sulfate, followed by trypsinization to remove cells. The cells were collected by centrifugation at 700 g at 4ºC for 3 min and suspended in 200 µL of Cell Scrub buffer (Genlantis Inc., San Diego, Calif., USA) to remove carriers bound to the surface of the cell membranes. After shaking at 4°C for 15 min, the cell suspension was centrifuged at 700 g for 3 min at 4ºC, and the pelleted fraction was resuspended in 500 µL of mitochondrial isolation buffer (MIB) [250mM sucrose, 2 mM Tris–HCl, 1 mM EDTA, pH 7.4]. The resulting suspension was homogenized by passage through a 27-gauge needle and centrifuged at 700 g for 10 min at 4ºC to remove the fraction containing nuclei and fracted cells. The resulting supernatant containing mitochondria was treated with RNase to remove RNA from the exterior of the mitochondria and centrifuged at 700 g for 10 min at 4ºC. The resulting supernatant was added onto a 60% percoll solution (GE Healthcare UK, Ltd., Buckinghamshire, UK) and centrifuged at 20,400g for 10 min at 4°C. The mitochondria present on the interface between the percoll solution and MIB were collected and centrifuged at 20,400 g for 15 min at 4°C. After removing the supernatant, the pelleted fraction was resuspended in 500 µL of EDTA-free MIB [250mM sucrose, 2 mM Tris–HCl, pH 7.4], and centrifuged at 20,400 g for 15 min at 4°C. The supernatant was removed to give the mitochondrial fraction. Total RNAs were extracted from the isolated mitochondria with an RNeasy Mini Kit (Qiagen, Hilden, Germany) according to the manufacture's protocol, combined with DNase I digestion for the degradation of DNA in total RNA samples using RNase-Free DNase Set (QIAGEN). The resulting RNA suspension was reverse transcribed using a High Capacity RNA-to-cDNA kit (Thermo fishier Scientific (Applied Biosystems)), according to the manufacturer's protocol. A quantitative ARMS-PCR analysis was performed on the cDNA, as described in the main text.

Supplementary Figures

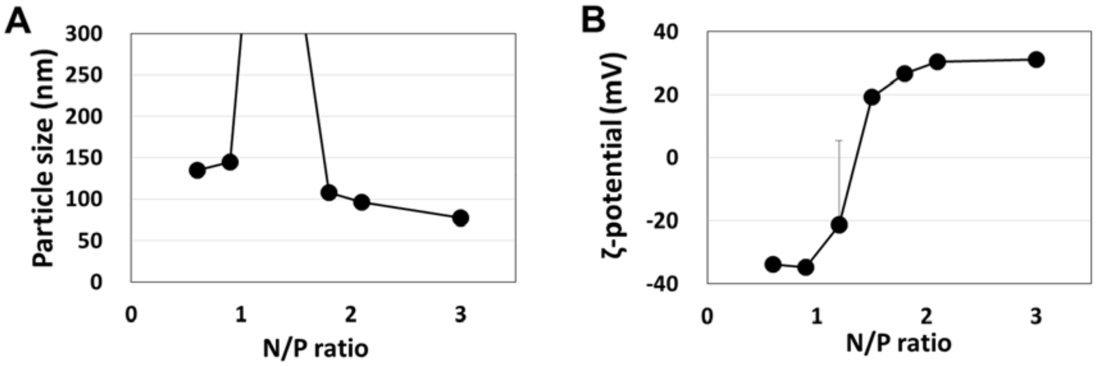

Figure S1 Diameters (A) and zeta (ζ) potentials (B) of the condensed mRNA particles that were prepared using protamine for a series of nitrogen/phosphate (N/P) ratios. Bars represent the mean ± S.D (n = 3).

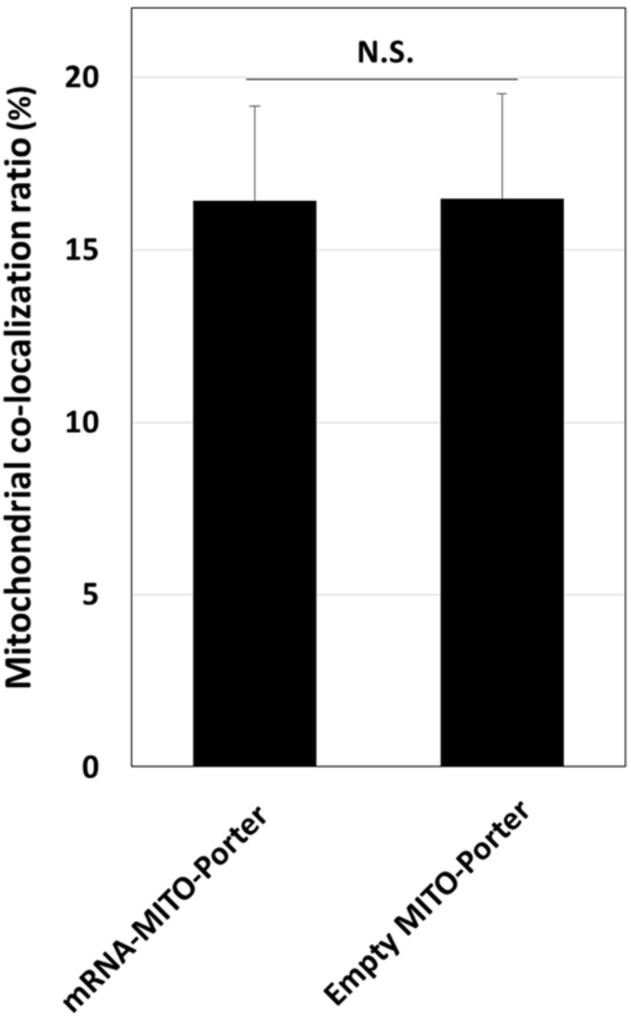

Figure S2 Quantification of the co-localization of MITO-Porter with mitochondria. Based on the images of the intracellular observation of mRNA-MITO-Porter or empty MITO-Porter, mitochondrial co-localization ratio was estimated. Mitochondrial co-localization ratio (%) per cell was calculated as follows: The number of co-localized particles (yellow) between mitochondria (red) and MITO-Porters (green)/ the total number of particles including co-localized particles (yellow) and non co-localized particles (green) x 100. Data are represented as the mean ± S.E. (n = 16-20). N.S., not significant deference.

Supplementary Vector sequences

The DNA vectors were designed by inserting the DNA fragment gene as shown below (**Sequences S1-S2**) into pUC57-Amp vector without promoter (GENEWIZ) between the multi cloning site (*EcoR* I and *EcoR* V sites).

Sequence S1 for pT7-WT-mRNA (ND3). The DNA sequence contains T7 promoter [highlighted in yellow], the sequence corresponding to artificial WT-mRNA (ND3) gene [highlighted in light green] with poly A sequence [highlighted in pink]. The T7 promoter was derived from pBluescript II SK (+) (Stratagene) to bases 626-644. The artificial WT-mRNA (ND3) gene was derived from human mtDNA corresponding to bases 10059-10404 (GenBank: NC_012920.1). The *T* shown by *italic* UPPER CASE letter is corresponding to base 10158 in mtDNA coding mRNA (ND3). Underlined letters indicate start codon and double underlined letters indicate stop codon.

*(5' EcoR*I*)* –
taatacgactcactatagggatGaacttcgccttaattttaataatcaacaccctcctagccttactactaataattattacattttga
ctaccacaactcaacggctacatagaaaaa*T*ccacccccttacgagtgcggcttcgaccctatatcccccgcccgcgtccctttc
tccataaaattcttcttagtagctattaccttcttattatttgatctagaaattgccctccttttacccctaccatgagccctacaaacaa
ctaacctgccactaatagttatgtcatccctcttattaatcatcatcctagccctaagtctggcctatgagtgactacaaaaaggatt
agactgaaccgaattAAaaaaaaaaaaaaaaaaaaaaaaaaaaaaaaaaaaaaaaaaaaaaaaaaaaaa - *(3' Eco* RV*)*

Sequence S2 for pT7-MT-mRNA (ND3). The DNA sequence contains T7 promoter [highlighted in yellow], the sequence corresponding to artificial MT-mRNA (ND3) gene [highlighted in light green] with poly A sequence [highlighted in pink]. The T7 promoter was derived from pBluescript II SK (+) (Stratagene) to bases 626-644. Mutant type mRNA (ND3) gene was synthesized by inserting a point mutation (T10158C), shown in an *italic* UPPER CASE letter (*C*) into the WT-mRNA (ND3). Under lined letters indicate start codon and double under lined letters indicate stop codon.

*(5' EcoR*I*)* –

taatacgactcactatagggatGaacttcgccttaattttaataatcaacaccctcctagccttactactaataattattacattttga
ctaccacaactcaacggctacatagaaaaa*C*ccacccccttacgagtgcggcttcgaccctatatcccccgcccgcgtccctttt
ctccataaaattcttcttagtagctattaccttcttattatttgatctagaaattgccctccttttacccctaccatgagccctacaaaca
actaacctgccactaatagttatgtcatccctcttattaatcatcatcctagccctaagtctggcctatgagtgactacaaaaaggat
tagactgaaccgaattAAaaaaaaaaaaaaaaaaaaaaaaaaaaaaaaaaaaaaaaaaaaaaaaaaaa

- *(3' Eco* RV*)*
